# Machine Learning Algorithms in EEG Analysis of Kleefstra Syndrome: Current Evidence and Future Directions

**DOI:** 10.3390/s25113420

**Published:** 2025-05-29

**Authors:** Katerina D. Tzimourta

**Affiliations:** Laboratory of Biomedical Technology and Digital Health, Department of Electrical and Computer Engineering, University of Western Macedonia, 50100 Kozani, Greece; ktzimourta@uowm.gr

**Keywords:** Kleefstra syndrome, electroencephalography, machine learning, EEG, biomarkers, rare neurodevelopmental disorders, 9q34.3, EHMT1, artificial intelligence

## Abstract

Kleefstra syndrome (KS) is a rare neurodevelopmental disorder associated with disruptions in the EHMT1 gene, often leading to intellectual disability, autism spectrum behaviors and epilepsy. The electroencephalogram (EEG) serves as a non-invasive tool to explore brain function in KS; yet, systematic characterizations of EEG features remain extremely limited. This review synthesizes current evidence on EEG findings in KS, highlighting the high prevalence of nonspecific abnormalities and seizures, but the absence of a consistent electrophysiological biomarker. Given the growing role of machine learning (ML) in extracting patterns from EEG data in related disorders—such as Angelman, Rett and Fragile X syndromes—this review explores how similar approaches could be adapted for KS. Despite promising perspectives, a lack of large-scale, publicly available EEG datasets hinders the application of ML methodologies in KS research. Future directions are proposed to address these gaps, including standardized EEG data collection, adoption of quantitative EEG analyses and integration of ML techniques adapted for small datasets. This multidisciplinary strategy holds potential for improving early diagnosis, monitoring and personalized interventions in Kleefstra syndrome.

## 1. Introduction

Kleefstra syndrome (KS) is a rare neurodevelopmental disorder caused by either a 9q34.3 terminal microdeletion or loss-of-function variants in the EHMT1 gene [1]. Clinically, KS is characterized by global developmental delay, moderate to severe intellectual disability (ID), childhood hypotonia, severely delayed or absent speech [2] and distinctive facial dysmorphisms [1,3,4]. Behavioral features can include autism spectrum disorder traits and, in adolescence or adulthood, episodes of developmental regression or psychiatric symptoms [5]. Given the neurological involvement, electroencephalography (EEG) has been an important tool to investigate brain function in KS, especially since a subset of patients experience seizures or other EEG abnormalities [6].

Several case series and clinical reports indicate that epilepsy is a significant comorbidity in Kleefstra syndrome. Approximately 20–30% of individuals with KS have seizures [1,4,7,8]. Reported seizure types are heterogeneous, ranging from generalized tonic–clonic and absence seizures to complex partial (focal) seizures [1,3,4,8]. However, the systematic characterization of EEG findings in KS has been limited due to the rarity of the condition. Understanding EEG patterns in KS is clinically relevant not only for managing seizures but also for exploring neurophysiological biomarkers of the syndrome.

In parallel, the rise of machine learning (ML) techniques in biomedical engineering offers new avenues for analyzing EEG data. Advanced algorithms, including traditional classifiers and deep learning models, can potentially detect subtle EEG biomarkers of neurological disorders that elude human visual analysis [9,10]. For common neurologic and developmental conditions, ML applied to EEG has shown promise in early diagnosis (e.g., autism in infants [11]) and in predicting treatment response (e.g., Rett syndrome therapies [12]). Recent research emphasizes the relevance of excitation/inhibition (E/I) balance in brain disorders such as Kleefstra syndrome, linking synaptic dysfunction to EEG alterations, as highlighted by BRAINMODEL studies [13]. For KS and similar rare genetic syndromes, machine learning could be transformative—from aiding diagnosis (especially in resource-limited settings where genetic testing is delayed) to tracking disease progression or treatment effects.

This paper presents a literature review of the relationship between Kleefstra syndrome, EEG findings and machine learning applications. We summarize known EEG abnormalities in KS across age groups (with emphasis on children), survey available datasets or studies of EEG in KS and comparable rare disorders, review how machine learning has been used to analyze such EEG data and identify gaps in knowledge. The aim is to provide a comprehensive technological and biomedical context for future research in this niche.

## 2. Materials and Methods

This section outlines the methodologies reported in the literature for analyzing EEG in Kleefstra syndrome and similar disorders, with an emphasis on technological and analytical approaches. Given that dedicated studies on KS are few, we draw on how researchers have collected, processed and analyzed EEG data in small-sample rare disorder studies and how machine learning techniques have been employed in comparable contexts.

To perform this review, we conducted a search of peer-reviewed publications in databases including PubMed, IEEE Xplore and Scopus, using keywords such as “Kleefstra syndrome” or “EHMT1” or “9q34.3” and “EEG” OR “electroencephalogram” and “machine learning” or “artificial intelligence”. We prioritized studies published in the last ~15 years (given that KS was first defined in 2005–2006) and focused on those that either described EEG findings in KS or applied quantitative analysis/machine learning to EEG in analogous pediatric neurodevelopmental disorders. A total of N = 15 primary articles were selected, including clinical case series, review papers and methodological studies. Data from patient advocacy resources and research registries were also included to supplement gaps in formal literature. No review protocol was registered for this study.

To arrive at the final corpus of 15 full-text papers, a structured search across different platforms was conducted. First, we ran the query (“Kleefstra syndrome” OR “EHMT1” OR “9q34.3”) AND (“EEG” OR “electroencephalogram”) AND (“machine learning” OR “artificial intelligence”) in PubMed, Scopus and IEEE Xplore, restricting the timespan to 2006–present, and this yielded 79 records. After automatic and manual de-duplication, 34 items were removed, leaving 45 unique titles/abstracts for screening. Five review articles that contained no primary EEG data were excluded at this stage. The remaining 40 full texts were assessed for eligibility; 35 were excluded for one of two reasons:

(i) molecular-genetic or clinical reports with no EEG data (n = 18);
(ii) animal-only studies (n = 7).

This sequential filtering produced the final set of 15 peer-reviewed articles that constitute the evidence base analyzed in this review.

## 3. Results

### 3.1. EEG Abnormalities in Kleefstra Syndrome

The literature consistently indicates that a significant minority of KS patients have abnormal EEGs or epilepsy. In GeneReviews and cohort studies, approximately one-third of KS individuals (around 25–30%) are reported to develop seizures [3,7]. These seizures often manifest in early childhood (sometimes in infancy) and can be diverse in presentation. A range of seizure types has been documented, including generalized tonic–clonic seizures, absence seizures and complex partial (focal) seizures [4,5,7,14]. Notably, a recent multicenter study [1] focusing on eight KS patients with epilepsy found that focal seizures were the most common manifestation [1]. In that cohort, all patients had moderate to severe developmental delays and hypotonia, and those with earlier seizure onset (before 3 years of age) tended to have more frequent seizures [1]. Over time, some patients experienced a decrease in seizure frequency and could reduce anti-seizure medications, suggesting a possible age-related improvement [1]. In the GenIDA registry study of 172 individuals, Zdolšek Draksler et al. [4] found that 39 respondents (25%) reported seizures, closely matching the 20–30% prevalence cited above. Absence and generalized tonic–clonic seizures each accounted for 9% of cases, while febrile convulsions and focal (complex-partial) seizures were reported by 6.4%. Two-thirds of those with epilepsy required continuous anti-seizure medication; valproate, levetiracetam and lamotrigine were the most frequently named agents, and 88% of caregivers rated treatment efficacy as “good” or “very good”. Only 13% described pharmacoresistant epilepsy, and one case employed vagus nerve stimulation. Despite these observations, epilepsy is a frequent finding in KS, but the underlying pathogenetic mechanism and specific features remain elusive [1]. In other words, while epilepsy is clearly part of the KS phenotype, no unique epileptic signature for KS has been confirmed to date.

Beyond overt seizures, KS individuals may exhibit EEG abnormalities even in the absence of clinical epilepsy. Caregivers and clinicians have noted instances of “abnormal EEG” in KS children who do not meet the threshold for a diagnosed seizure disorder [2]. These abnormalities can include nonspecific findings such as focal sharp waves or a slow/disorganized background rhythm. For example, a case report of an 8-year-old boy with KS and sleep disturbances documented that an overnight EEG showed intermittent sharp wave anomalies in the right hemisphere [5]. Similarly, patient registry data and caregiver reports (e.g., via the IDefine [15] and IDefine Europe [16] KS community) list “seizures or abnormal EEG” as one of the neurological indicators of Kleefstra syndrome [2]. This implies that clinicians often encounter EEG deviations in KS patients, though they may not always manifest as clinical seizures.

It is important to emphasize that no pathognomonic EEG pattern has been established for Kleefstra syndrome. This is analogous to many other rare neurogenetic syndromes: for instance, KBG syndrome (caused by ANKRD11 mutations) also shows a high rate of EEG anomalies without a distinct syndrome-specific pattern [17]. A recent comprehensive review on epilepsy in chromosomal microrearrangement syndromes noted that, to date, “no peculiar interictal electroencephalographic patterns have been identified” in these conditions [12]. KS falls into this category; unlike Angelman syndrome, which often exhibits a characteristic rhythmic 2–3 Hz delta with frontal notches on EEG [12], or Rett syndrome which has a recognized progression of EEG changes with age [18], Kleefstra syndrome’s EEG findings are comparatively nonspecific. The background EEG in KS may be diffusely slow, consistent with developmental impairment, and interictal epileptiform discharges (spikes or sharp waves) can occur, but their location and frequency vary among individuals. Some KS patients have completely normal EEGs [3,19,20], especially at younger ages or prior to any seizure onset [20]. Given this variability, clinicians currently rely on general epilepsy evaluations (e.g., routine EEG or overnight EEG if needed) for KS patients with suspected seizures, rather than looking for a unique KS EEG signature. Alterations in excitation/inhibition ratio at the neuronal level, as discussed in recent research [13], may partly explain the heterogeneous EEG findings observed in Kleefstra syndrome.

For KS individuals without clinical seizures, EEG can range from normal to showing nonspecific anomalies. Some children have entirely normal EEG recordings (especially at young ages before any potential epilepsy manifests) [20]. Others might show mild abnormalities such as intermittent slowing or an occasional spike in an otherwise asymptomatic child [17]. A notable insight from a related syndrome (KBG) is that nonspecific EEG anomalies were seen in up to 72% of patients even if only ~25% had definite seizures [17]. Although equivalent statistics are not published for KS, it is reasonable to suspect a similar pattern: many KS patients could have minor EEG irregularities, reflecting the underlying brain dysfunction, even if they never develop recognizable epilepsy. These irregularities alone are typically not diagnostic—they overlap with EEG findings in other forms of intellectual disability. Thus, the current clinical practice for KS is to use EEG primarily as a diagnostic tool for suspected seizures or regression, rather than as a screening tool for the syndrome. GeneReviews accordingly recommends EEG in KS “if seizures are suspected” [7] and does not list any EEG trait as a diagnostic criterion for the syndrome. Table 1 summarizes the key EEG phenotypes reported in Kleefstra syndrome, detailing their electrographic features, clinical context and qualitative frequency.

### 3.2. EEG Data Collection and Processing in Kleefstra Syndrome

In the reviewed KS case series, EEG recordings were typically obtained as standard clinical evaluations. For instance, Giacomini et al. [1] performed routine video-EEG monitoring or prolonged EEG in their eight KS patients with epilepsy, capturing both wakefulness and sleep to observe interictal discharges [1]. EEG was recorded using the international 10–20 system of electrode placement (common in clinical EEG). Similarly, earlier case reports document that EEGs were performed in children awake or during sleep if seizures or sleep issues were reported [5]. The methodological challenge is that each KS patient may have had EEG at different ages and with different protocols, making it hard to aggregate data. None of the KS-specific sources reported using a standardized research EEG protocol (such as a fixed-length resting-state EEG with eyes closed/open). Therefore, one methodological need is for harmonized EEG acquisition in any future multi-patient KS study—possibly acquiring a short resting-state EEG from all participants under similar conditions, to allow comparison.

Traditionally, EEGs in KS have been analyzed qualitatively by neurologists, who were looking for spikes, sharp waves or slowing. For example, in the reported KS epilepsy cases, the authors qualitatively noted whether seizures were focal (with corresponding focal EEG onsets) or generalized and whether interictal EEG showed focal epileptiform abnormalities [1]. There was no mention of specialized quantitative analyses in those reports. However, learning from other disorders, there is a push towards quantitative EEG (qEEG) metrics [21]. In the context of rare neurodevelopmental disorders, qEEG methods included spectral power analysis, functional connectivity estimation (e.g., coherence or phase–amplitude coupling between EEG channels) and aperiodic component analysis (extracting the 1/f background slope) [12,22]. For instance, Dubois et al. [22] computed power spectral density and network connectivity for each subject’s EEG and then compared those metrics between CNV carriers and controls after correcting for age [22]. This approach revealed subtle differences (like altered alpha frequencies) that might not be apparent by eye. Methodologically, such analyses require clean EEG data and often multiple artifact-free epochs. In pediatric populations, artifact removal (due to movement, etc.) is a key step, and techniques like independent component analysis (ICA) or automated artifact rejection algorithms are often employed to preprocess EEG before computing features [23].

If applied to Kleefstra syndrome, similar qEEG processing pipelines would be recommended. We expect steps would include high-pass filtering (to remove drift), removal of line noise, ICA to remove eye-blinks or muscle noise, segmentation into epochs and feature extraction (power in delta, theta, alpha, beta, gamma bands; global vs regional measures; etc.). Given the likely small N, statistical comparison of such features would use non-parametric tests or bootstrap methods to assess significance.

### 3.3. EEG Datasets and Studies in Rare Disorders

Due to the rarity of Kleefstra syndrome (estimated prevalence ~1 in 200,000 [5]), there are no known publicly available EEG datasets dedicated to this condition. Most published KS EEG findings come from small cohorts or single-case reports, and the EEG data are not shared in open repositories. This lack of open data poses two major challenges: (1) it limits the statistical power and generalizability of existing findings and (2) it hinders the application of advanced analytic tools, such as machine learning, which typically require larger sample sizes.

For instance, the eight patients in the multicenter study by Giacomini et al. [1] had their EEGs analyzed for research purposes, but only aggregated findings are reported. Similarly, no longitudinal EEG studies have been published to date. However, research registries are starting to address this gap. The Simons Searchlight program [24] includes EHMT1-related syndromes in its rare disorder registry and has collected clinical and neurophysiological information, such as the presence of seizures, in a small KS cohort. As of a recent update, ~5 out of 13 KS participants had a history of seizures (≈38%), and many had hypotonia (100%) [24]. Although raw EEG data are not yet available, such efforts could eventually facilitate large-scale collaborative studies by connecting researchers with willing KS families.

This direction reflects a broader trend. Patient advocacy and research networks are starting to emphasize data sharing for neurogenetic disorders. One example is the SLC6A1 gene disorder community (a different rare epilepsy syndrome): the SLC6A1 Connect foundation recently proposed creating an anonymized public EEG dataset labeled with genetic diagnoses [10]. Their goal is to enable AI researchers to discover EEG biomarkers for rare genetic epilepsies, which could aid earlier diagnosis [10]. This underscores a broader trend—while currently each rare disorder has sparse data, combining EEG data across comparable conditions or multicenter collaborations could yield sufficiently large datasets. In the case of KS, data pooling through registries is slowly progressing.

Beyond KS, a few studies have looked at EEG in comparable rare neurodevelopmental disorders. For example, a 2025 study by Dubois et al. examined resting-state EEG in 109 children with various pathogenic copy number variations (CNVs) associated with neurodevelopmental disorders genetic [22]. Although Kleefstra syndrome was not explicitly included, that study found that children carrying large genomic deletions or duplications showed altered EEG spectral power and connectivity compared to controls—notably a reduced alpha peak frequency and differences in aperiodic (1/f) activity [22]. Interestingly, the EEG deviations were present regardless of the specific genes involved, suggesting that many neurogenetic disorders have convergent effects on brain rhythms [22]. Such findings hint that if enough KS patients were studied, they might also show measurable quantitative EEG differences (e.g., in power spectra or network coherence) relative to neurotypical children.

In contrast to the limited EEG literature on KS, other syndromes such as Angelman and Rett have been more extensively studied. Typically, children with Angelman syndrome exhibit high-amplitude rhythmic activity in the 4–6 Hz range over posterior brain regions, often accompanied by spike components and accentuated by eye closure [12,25]. Additionally, a prominent 2–3 Hz high-amplitude rhythmic activity over frontal areas, sometimes with epileptiform discharges, is frequently observed in both children and adults with this syndrome. These characteristic EEG patterns, which include delta and theta rhythms as well as posterior discharges, often coexist and can aid in early diagnosis even before the full clinical symptoms develop. In contrast, Rett syndrome (MECP2 mutations) exhibits an age-dependent evolution: early childhood EEGs may be normal or display focal centro-temporal spikes, but by mid-childhood, generalized slowing emerges, followed by diffuse spike–wave or burst–suppression patterns in adolescence, mirroring clinical regression [26]. Rett EEG is dominated by a diffuse low-frequency background plus a sensorimotor rhythm that shifts to unusually low frequencies and loses its upper-band power. These two quantitative measures—the amplitude of generalized slowing and the μ frequency rate index—track overall RSS severity and specific symptom clusters (motor impairment, speech loss, breathing dysrhythmia) better than raw spike counts [27].

Furthermore, the different age distribution of participants can significantly affect the results of EEG analysis, as EEG features undergo substantial developmental changes throughout childhood and adolescence. It is well established that absolute EEG power, particularly in slow-wave frequencies such as delta and theta bands, decreases linearly with increasing age in typically developing children, reflecting maturational processes like synaptic pruning and cortical reorganization [28]. This age-related decline in EEG power has also been observed in neurodevelopmental disorders such as ADHD, where the developmental trajectory parallels but differs in magnitude from that of healthy controls [28]. Therefore, when analyzing EEG data in heterogeneous clinical populations, differences in age distribution must be carefully accounted for, as they can confound group comparisons and obscure disorder-specific EEG signatures [29]. Future studies should incorporate age as a continuous covariate or use age-prediction modeling to better isolate pathological EEG alterations from normative developmental changes, thereby enhancing the precision and interpretability of EEG findings in neurodevelopmental disorders.

### 3.4. The BRAINMODEL Project

BRAINMODEL is a Dutch consortium launched in 2021 with the goal of improving personalized treatment for neurodevelopmental disorders by linking insights “from bench to bedside” [30]. It brings together six research institutions with a EUR 4 million grant to develop new methods for translating molecular and cellular findings into clinical applications [13,30]. A central focus of BRAINMODEL is on monogenic neurodevelopmental syndromes, including Kleefstra syndrome, as paradigms to study brain dysfunction. Kleefstra syndrome (caused by EHMT1 haploinsufficiency) is one of the project’s exemplar “chromatinopathies”, alongside certain synaptic disorders (“SNAREopathies”) [30]. The consortium’s multi-level strategy involves deriving patient-specific induced pluripotent stem cells (iPSC) and growing them into neuronal networks in vitro, while recording clinical EEG from the same patients to identify translational biomarkers [30]. The aim is to link cellular excitation/inhibition (E/I) imbalances to EEG phenotypes and patient-reported outcomes, ultimately informing personalized therapies [30]. In their foundational paper, Geertjens et al. [13] outline this approach, emphasizing that novel EEG measurements in patients will be tied to the cellular models to maintain E/I ratio homeostasis across experimental levels [30].

Within BRAINMODEL, Kleefstra syndrome features prominently: the project is co-led by experts in Kleefstra syndrome (including Dr. Tjitske Kleefstra) and has engaged the patient community. As of early 2023, BRAINMODEL’s clinical study began recruiting children, successfully obtaining ethics approval and enrolling at least nine patients [30]. Each participant undergoes comprehensive assessments (medical, behavioral, metabolic, immune) and an EEG recording of brain activity and, for a subset, researchers culture the patient’s own stem cells into neurons for parallel in vitro analysis. This ambitious design will enable direct comparisons between a child’s EEG features and their neuronal cell biology, providing unprecedented insight into how a genetic mutation (like EHMT1 in Kleefstra syndrome) leads to network-level dysfunction. While BRAINMODEL is still in progress with no published patient EEG results yet, its proof-of-concept rests on prior findings that patient-derived neurons can recapitulate network deficits. Indeed, earlier work in Kleefstra syndrome iPSC models showed aberrant neural network bursting and NMDA receptor hyperactivity, suggesting an excitatory/inhibitory imbalance that might also be detectable in patient EEG [31].

### 3.5. COMBINEDBrain Consortium

On an international scale, the COMBINEDBrain consortium represents a major initiative to pool data and expertise across many rare genetic neurodevelopmental disorders. COMBINEDBrain (Consortium for Outcome Measures and Biomarkers for Neurodevelopmental Disorders) was established in 2019 as a non-profit alliance of over 30 patient advocacy groups, along with clinicians, scientists and industry partners [32]. Its mission is to “speed the path to clinical treatments for people with rare genetic neurological disorders by pooling efforts, studies and data” [32]. A key strategy of COMBINEDBrain is to develop and share outcome measures and biomarkers that can be used in clinical trials across different syndromes. EEG-based biomarkers are a particular area of interest, given EEG’s sensitivity to brain network function. Member organizations in COMBINEDBrain include those devoted to Kleefstra syndrome (the IDefine Foundation [15]) alongside many others (e.g., SYNGAP1, CDKL5, ADNP, etc.), enabling data sharing for otherwise ultra-rare conditions [33]. Through this consortium, families and researchers collaborate on “disease concept studies” to define the phenotype of each disorder in depth, often incorporating EEG assessments to characterize patterns of brain activity. For example, the Kleefstra syndrome community, via IDefine and COMBINEDBrain, has launched a biorepository and data collection effort that will support research into EEG and other biomarkers [6]. Similarly, the GRIN2B Foundation (another COMBINEDBrain member) funded a project by Dr. Caitlin Hudac to collect EEG during sensory processing tasks in GRIN2B-related disorder patients, aiming to develop novel EEG-based biomarkers that link brain activity with behavior. By aggregating multicenter data, COMBINEDBrain also facilitates machine learning analyses: large, pooled EEG datasets can be used to train algorithms to detect abnormal network signatures or predict clinical outcomes. While consortium-wide results are just beginning to emerge, this collaborative model has already yielded valuable resources (shared data, common protocols) and strategic roadmaps for therapy development. Notably, COMBINEDBrain’s approach was cited as a model in a recent CHD2 syndrome research roadmap, alongside the Rare Epilepsy Network, for its role in accelerating biomarker discovery in rare epileptic encephalopathies [34]. As COMBINEDBrain continues to grow, we anticipate publications that leverage its pooled EEG data to define common neural phenotypes and test ML-driven diagnostic tools across multiple rare syndromes.

## 4. Discussion

### 4.1. Comparative Data and Methodologies from Other Rare Syndromes

Insights from EEG studies in analogous neurodevelopmental syndromes can provide context. For example, Angelman, Rett and Fragile X syndromes—while genetically different from KS—illustrate how EEG can reflect underlying neurobiological differences. In Angelman syndrome (UBE3A mutations), >80% of patients show intermittent rhythmic delta activity with frontal notching and ~75% show epileptiform discharges [12]. In Rett syndrome (MECP2 mutations), EEG evolves through stages from normal to early focal epileptiform activity and later generalized slowing and spikes [18]. These EEG signatures have even been proposed as biomarkers for severity in those disorders [12]. By contrast, Kleefstra syndrome lacks a well-defined EEG biomarker. A few case reports note features like centrotemporal spikes or temporal sharp waves in some KS patients (resembling patterns seen in benign focal epilepsy of childhood), but these are not consistent across the cohort. The heterogeneity of genetic mutations (different sizes of 9q34 deletions or various EHMT1 point mutations) and the relatively small numbers studied make it challenging to identify subtle commonalities. Thus, current literature suggests that any EEG abnormalities in KS are generally nonspecific and primarily serve to document the presence of seizures or encephalopathic activity, rather than to diagnose KS itself.

We also note how some rare disorder studies combined multimodal data. In KBG syndrome (which, like KS, lacks a known EEG marker), researchers have suggested using AI-driven phenotyping by combining facial recognition AI (for dysmorphic features) with EEG screening findings [17]. The idea is that an integrated tool could flag a possible syndrome if both face analysis and EEG show certain patterns. While still speculative, it highlights a methodological trend: leveraging multiple data sources (imaging, EEG, speech [2], genomic data) in machine learning models to improve diagnostic power for rare diseases [10]. A parallel effort already exists for Kleefstra syndrome: the University of Bonn’s GestaltMatcher group has trained deep learning models to recognize the characteristic KS facial gestalt and is actively validating them in international cohorts [35]. For KS, a future approach could integrate EEG features with, say, the distinctive facial features or with other biomarkers (like the recently discovered blood DNA methylation signature of KS [7]), using ML to weigh the evidence from each modality.

### 4.2. Machine Learning in EEG Analysis for Rare Disorders

In general, pediatric neurology, Random Forests and similar classifiers have been evaluated in order to detect or predict neurological disorders from EEG [29,36]. Research has also applied ML to EEG-based seizure detection and phenotyping, which is very relevant to KS, given the prevalence of epilepsy. In general epilepsy studies, deep learning models like CNNs and recurrent neural networks (RNNs) have become popular for automated seizure detection in EEG recordings [29,36]. These models learn to recognize the complex waveform patterns preceding or during a seizure.

Although KS patients typically have focal seizures, algorithms trained on large EEG datasets (such as the TUH [37] or CHB-MIT scalp EEG database [38]) could potentially be fine-tuned on KS-specific data to improve detection of subtle seizures or epileptiform discharges in this population. Moreover, if KS had any subclinical EEG signature, a sufficiently sensitive ML model might detect it. However, techniques for small and imbalanced data in EEG analytics are an active research area. Approaches such as data augmentation, transfer learning from larger EEG datasets and one-class or outlier detection algorithms have been proposed to tackle scenarios with few patients [39]. These methods could be harnessed to enable ML on rare disease EEG data. Additionally, signal compression techniques—such as adaptive stepsize forward–backward pursuit (AS-FBP)—may offer advantages in reducing training time and memory usage when processing high-dimensional EEG data, although their application in rare neurodevelopmental disorders remains unexplored [40]. Moreover, the incorporation of EEG datasets currently being collected through large-scale collaborative initiatives—such as the BRAINMODEL project and the COMBINEDBrain consortium—could substantially enhance this effort. These consortia aim to harmonize EEG acquisition protocols across syndromes and promote open data sharing, ultimately facilitating robust ML model training, reducing overfitting risks and improving the generalization of EEG-based biomarkers in Kleefstra syndrome and other rare neurodevelopmental disorders.

Finally, machine learning has shown value in quantitative EEG (qEEG) biomarker discovery for neurodevelopmental disorders. Instead of classification, some studies use ML to find patterns that correlate with symptom severity or treatment response. For example, in a clinical trial for Rett syndrome, researchers computed EEG network features (connectivity graphs) for each participant before treatment. They reported that a ML model using these pretreatment network features could predict which patients would respond to an experimental therapy with 100% accuracy in their sample [41]. Similarly, in Angelman syndrome, a study of 45 patients applied Support Vector Regression to delta spectral power and found a strong correlation with clinical severity [42]. In Fragile X syndrome, Ethridge et al. [43] applied a multivariate ML model including spectral and complexity measures, achieving an AUC of 0.9610 for distinguishing patients from controls—demonstrating robust diagnostic potential. In contrast, disorders like KBG and Kleefstra syndrome currently lack both well-defined EEG biomarkers and ML-based EEG studies. For KS, which currently has no specific treatments beyond supportive care, such analyses might in the future stratify patients by severity or monitor changes. By analogy, one could envision training an algorithm to distinguish KS patients from typical controls via EEG features, if enough data were available. Table 2 provides a brief overview of EEG abnormalities, the presence of EEG signatures and the application of ML in the selected rare neurodevelopmental syndromes.

### 4.3. Applications of Machine Learning—Current State

This review indicates that direct applications of machine learning to Kleefstra EEG data have not been reported as of April 2025. This is not surprising, given the data limitation noted above. Nonetheless, we glean insights from analogous applications in other disorders: they show proof-of-concept that ML could extract valuable information from EEG in rare disorders. Key findings from related work include the following:

- **Diagnostic Classification:** Machine learning can classify patient vs control EEGs with high accuracy in certain conditions. In the Bosl et al. autism study [11] for example, researchers computed nonlinear EEG features (e.g., fractal dimensions, entropies) for each infant and then fed these into algorithms like support vector machines (SVM) to predict outcome [11]. They tried k-nearest neighbors, random forests and SVM, ultimately using an SVM with an RBF kernel, which achieved >95% accuracy in distinguishing ASD vs non-ASD based on EEG [11]. While ASD is more common than KS, the principle holds that early brain functional differences can be detected via ML. In rare genetic syndromes, one could envision a tool that analyzes a child’s EEG and outputs a probability of a specific syndrome (e.g., KS vs Angelman vs others) to prompt targeted genetic testing. In fact, patient advocates have suggested using AI on routine EEGs as a screening proxy for genetic testing in undiagnosed children [10]. Such an approach is in its infancy, but the conceptual feasibility is supported by results in more studied conditions (e.g., distinguishing schizophrenia patients by EEG patterns via deep learning [10].
- **Biomarker Discovery:** Quantitative EEG changes correlated with phenotype have been discovered through ML and advanced analysis. In Rett syndrome clinical trials, network analysis combined with machine learning was able to separate treatment responders from non-responders purely from pretreatment EEG, with 100% accuracy in one small study [41]. For KS, applying similar analyses could be highly informative. For instance, does a KS child with more pronounced EEG slowing have a more severe cognitive impairment? Are there EEG metrics that correlate with the degree of regression in adolescence? These questions remain unanswered. Filling this gap could not only improve clinical monitoring but also provide objective endpoints for any future therapeutic trials in KS (where EEG could serve as a measure of neurological improvement or stabilization).
- **Seizure Prediction and Management:** Outside of syndrome diagnosis, ML applied to EEG is extensively used in seizure detection/prediction. For KS patients with epilepsy, adapting those tools could improve care. No study specifically addresses KS seizure prediction, but given that most KS seizures are focal, one interesting avenue is the use of ML to detect the precursors of focal seizures (e.g., changes in EEG power or network topology minutes before a seizure). Modern algorithms can sometimes forecast seizures by recognizing subtle pre-ictal patterns. If KS caregivers were equipped with seizure prediction devices (e.g., wearable EEG with AI), it could enhance safety. This is speculative but highlights a potential area for contribution: customizing general epilepsy AI technology to the specific seizure profiles and EEG morphologies seen in KS.

Taken together, current literature demonstrates significant gaps at the intersection of KS, EEG and ML, specifically the following:

(1) There is no comprehensive description of EEG features in a large KS cohort—we lack knowledge of whether KS has a mild but consistent EEG signature (for example, do all KS patients show a slightly lower dominant frequency compared to peers, as CNV carriers did in one study? [22]).
(2) There are no public or large shared EEG datasets for KS, which slows research progress.
(3) Machine learning techniques, which could uncover hidden patterns in KS EEG, have not yet been applied, leaving a rich potential unexploited.
(4) Because of these, clinicians currently do not use EEG for anything beyond conventional seizure management in KS—unlike some other syndromes where EEG might aid earlier diagnosis (e.g., the notched delta in Angelman can raise clinical suspicion of that diagnosis [12]).

### 4.4. Potential Areas for Future Contribution

**Figure 1:**
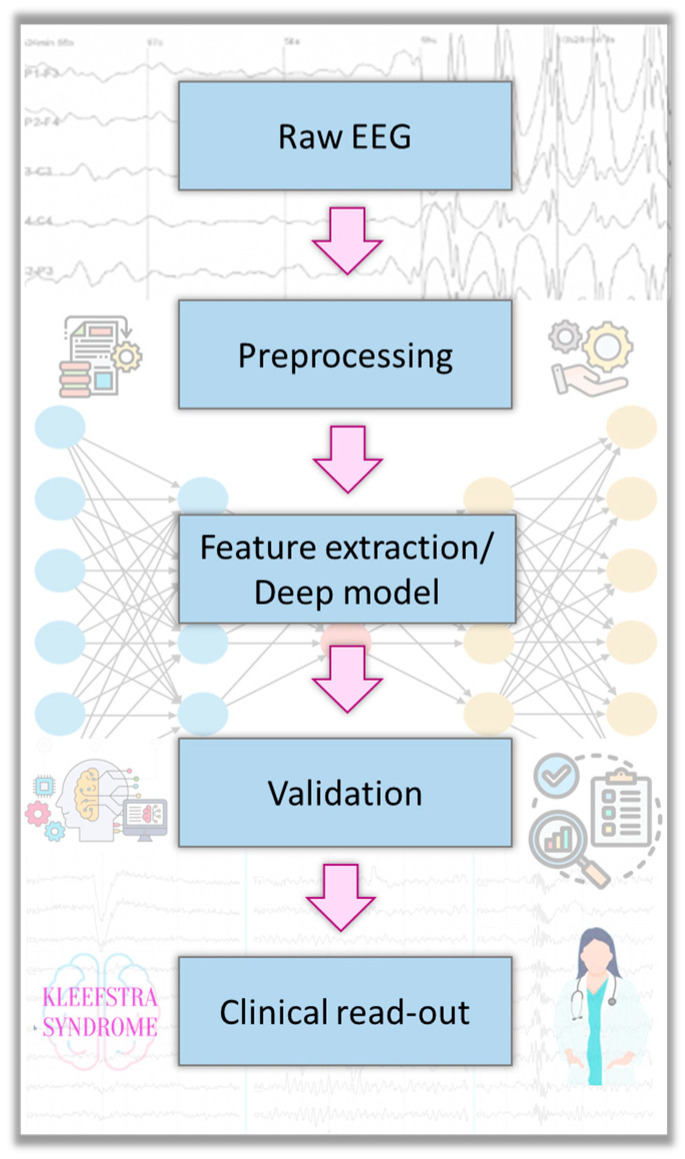
Proposed machine learning workflow for EEG analysis of Kleefstra syndrome.

A central focus is how machine learning has been or could be used to interpret EEGs in KS and related syndromes. Figure 1 depicts the proposed machine learning workflow for Kleefstra syndrome EEG analysis, from raw-signal preprocessing to a clinically interpretable output. The proposed workflow is derived by analogy and does not constitute fully validated protocols for KS. The methodologies can be broadly divided into two paradigms:

- **Feature-Based Machine Learning:** This involves manually extracting features from EEG signals and then using classical ML algorithms for classification or regression. In any future KS EEG study, one might extract features such as average slow-wave (delta) power, spike frequency or network synchrony metrics and then train a classifier to distinguish KS patients from other groups. Support vector machines and Random Forests are popular choices for such small-data classification problems because they can work well with limited samples and many features, especially with careful cross-validation to avoid overfitting. Other methods like logistic regression or Gaussian naive Bayes could also be tested as baselines. Feature selection or dimensionality reduction (e.g., principal component analysis) is often incorporated to handle the high-dimensional feature space relative to the number of subjects.
- **Deep Learning on EEG:** Deep learning models, particularly CNNs, have revolutionized EEG analysis in larger datasets, as they can learn complex spatiotemporal features directly from raw signals or from transformed representations (like time–frequency spectrograms). For rare disorders with small N, deep learning must be used cautiously to avoid overfitting. However, another methodology is transfer learning: using a CNN pre-trained on a big EEG dataset (for example, a seizure detection CNN trained on thousands of hours of EEG from an epilepsy database) and then fine-tuning it on the rare disorder EEG data to detect more subtle patterns. Another approach is data augmentation—creating synthetic EEG segments (perhaps using methods like GANs or by simple slicing/windowing techniques) to effectively increase the training set size. Although we did not find an example of CNN or RNN applied specifically to a rare syndrome EEG classification, these techniques are well documented in the epilepsy literature [29,44]. For instance, CNN-based algorithms have achieved impressive accuracy in identifying epileptic vs normal EEG segments and even differentiating seizure types. An RNN (such as an LSTM network) could capture temporal dynamics in KS EEG, like sequences of microstates or recurrent patterns over minutes. Hybrid models (combining CNN for spatial feature extraction and RNN for temporal modeling) have also been proposed for EEG analysis. The DICE-Net architecture (a convolution-transformer network) for Alzheimer’s EEG classification [23] exemplifies cutting-edge deep learning that could conceivably be adapted to detect subtle encephalopathic patterns in KS.
- **Validation and Evaluation:** For any ML methodology applied to EEG, rigorous validation is crucial. The literature emphasizes techniques such as k-fold cross-validation or leave-one-subject-out validation when sample sizes are small [45]. Performance metrics commonly reported include accuracy, sensitivity (recall), specificity and the area under the ROC curve (AUC). In an ASD prediction study, for instance, the model’s positive predictive value (PPV) and sensitivity were extremely high (>95%) when evaluated on held-out data at certain time points [11]. In a KS context, one might also consider unsupervised learning methods due to limited labeled data—for example, clustering algorithms to see if EEG feature profiles cluster KS patients separately from others, or anomaly detection algorithms that treat KS as “outliers” against a background of typical EEGs.

Table 3 presents a gap analysis that links current research limitations to recommended actions and the expected scientific or clinical impact. Based on these gaps, the review identifies several promising avenues for future research and contributions:

- Data Collection and Sharing: A foundational step is to gather a larger dataset of EEG recordings from individuals with Kleefstra syndrome. Multi-center collaborations or family-led registries could enable the collection of standardized resting-state and sleep EEG from dozens of KS patients. Importantly, making such a dataset publicly available (with proper anonymization and consent) would invite machine learning experts to contribute analyses [10]. As seen with other disorders, when data is shared, progress accelerates. The KS community, possibly through organizations like IDefine [15] and IDefine Europe [16] or partnerships with platforms like Rare-X and GestaltMatcher, could spearhead an “EEG data commons” for Kleefstra syndrome. This would directly address the current lack of data.
- Quantitative EEG Characterization: Even prior to complex ML, performing a thorough quantitative analysis of KS EEGs would be valuable. Future studies should compute spectral features, connectivity metrics and sleep microstructure (if overnight EEG or polysomnography is available) for KS cohorts and compare them to neurotypical controls or to individuals with other ID syndromes. This might reveal, for example, that KS brains have a particular profile of oscillatory activity. Any distinctive pattern, once confirmed, could become a biomarker. This is low-hanging fruit that modern EEG analysis software can accomplish, but it requires assembling the recordings first.
- Machine Learning Diagnostic Models: With enough data, researchers can train ML models to classify EEGs as KS or non-KS. Even if perfection is not attainable, a moderately accurate model could be useful as a screening tool. For instance, in an undiagnosed child with developmental delay, an “EEG ML panel” might output probabilities for several genetic syndromes (Kleefstra, Angelman, Tuberous Sclerosis, etc.) based on the EEG pattern. As highlighted by the SLC6A1 initiative, this could guide genetic testing earlier and shorten the diagnostic odyssey [10]. Developing such models for KS would contribute to precision medicine by leveraging an easily obtainable test (EEG) to hint at a genetic diagnosis.
- Prognostic and Monitoring Tools: Another potential contribution is using EEG-based ML to track disease progression or therapeutic response in KS. For example, if a future clinical trial tests a drug targeting EHMT1 pathways, EEG could be used to quantify brain function changes. ML could potentially be explored for KS patients, which could serve as an outcome measure. Additionally, longitudinal EEG data analyzed with ML might predict which children are at risk of neuropsychiatric regression in adolescence—a known concern in KS [5,7]. Early predictions could prompt proactive interventions.
- Cross-Disorder Insights: Finally, a broader contribution would be to integrate KS EEG findings into the larger map of rare neurodevelopmental disorders [46]. By applying uniform ML analyses across multiple syndromes (e.g., KS, KBG, Pitt–Hopkins, Phelan–McDermid, etc.), we can identify shared neural signatures or important differences. The recent CNV EEG study hinted that many disorders share EEG alterations despite different genetics [22]. Confirming this with more syndromes could shift the focus toward treating common circuit-level problems rather than each genetic disorder in isolation. For KS, contributing data to such comparative studies ensures it is included in the development of broadly effective neurotherapeutic strategies.

## 5. Conclusions

In conclusion, the results of this literature review highlight that Kleefstra syndrome presents a clear opportunity for interdisciplinary research. Neurologists and geneticists have outlined the clinical and EEG aspects of the syndrome on a basic level (seizure prevalence and types), but the fine-grained characterization and innovative analysis lag behind. In similar rare disorders, quantitative EEG analyses have revealed disease-specific signatures that correlate with developmental disability and seizure [12] burden. These findings underscore the potential of EEG biomarkers to reflect underlying pathophysiology and severity in rare disorders. By learning from successes in similar conditions and addressing current data gaps, future work can establish EEG-based biomarkers for Kleefstra syndrome and apply machine learning techniques to improve diagnosis, monitoring and, ultimately, patient outcomes. The marriage of EEG and AI in this rare disorder, though in early stages, holds promise for unlocking new understanding of KS’s impact on the brain [10] and perhaps even guiding interventions in the years to come.

## Figures and Tables

**Table 1:** Electroencephalographic phenotypes reported in Kleefstra syndrome. Summary of the main EEG patterns associated with clinical contexts and qualitative incidence as extracted from the primary studies included in this review.

EEG Phenotype	EEG Description	Clinical Context/Notes	Reported Incidence	Ref.
Focal epileptiform discharges	Spikes/sharp waves confined to one region (often centro- temporal, temporal, or frontal)	Interictal correlate of the predominantly focal seizures described in KS; occasionally seen in patients without overt epilepsy	Common in epileptic KS cohorts	[1,4,5,7]
Diffuse/ generalized background slowing	Globally reduced posterior dominant rhythm; disorganized or low-frequency background	Mirrors moderate-severe intellectual disability and hypotonia typical of KS	Recurrent finding across small series and case reports but not systematically quantified	[5,17]
Generalized epileptiform activity	Generalized spike–wave or polyspike–wave bursts	Generalized tonic–clonic or absence seizures	Occasional (isolated case descriptions)	[1,4,5,7]
Refractory epilepsy (Infantile Spasms/ Lennox–Gastaut pattern)	Generalized slow spike-and-wave with paroxysmal fast activity	Not syndrome-specific but noted in a minority of KS children with seizures	Rare (individual cases)	[4,8]
Normal EEG	No epileptiform discharges; age-appropriate background	More often in younger children or KS individuals without seizures	Subset of patients (exact proportion unknown)	[3,19,20]

**Table 2:** Summary of EEG abnormalities and machine learning applications across specific rare syndromes.

Syndrome	EEG Abnormalities	EEG Signature	Study (Year)	No. of Cases	ML Method	Results
Angelman	Rhythmic delta, frontal notch	Yes	Hip et al. (2021) [42]	45 patients	Delta Spectral Power, Linear Support Vector Regression	Delta power correlates with severity
Rett	Stage-dependent, slowing	Yes	Keogh et al. (2020) [41]	18 patients (9 treated and 9 non-treated)	Spectral Power, SVM	Accuracy: 100%
Fragile X	Increased resting gamma power	Yes	Ethridge et al. (2024) [43]	141 cases (70 patients 71 controls)	Spectral power, Alpha peak frequency, Theta/beta ratio, multi-scale entropy, naive Bayes	AUC = 0.9610
KGB	Nonspecific, frequent	No	Not reported	Not reported	Not reported	Not reported
Kleefstra	Focal sharp waves, slowing	No	Not reported	Not reported	Not reported	Not reported

**Table 3:** Gap analysis and recommended future directions for EEG + machine learning research in Kleefstra syndrome.

Domain	Current Gap	Proposed Action	Expected Impact
Data availability	No open, large-scale EEG repositories for KS; limited published studies	Launch multi-center “EEG Commons” Deposit raw scalp EEG plus minimal phenotypic metadata in BIDS-EEG format	Enables reproducible research; provides training data for ML models; accelerates biomarker discovery
Acquisition standards	Heterogeneous clinical recordings (different montages, durations, states) hamper pooling	Agree on core protocol: 10-min resting-state (eyes-open/closed) + overnight PSG where feasible; record sampling rate, state, meds	Harmonized signals → comparable quantitative features; reduces confounds in cross-site ML
Machine-learning use	No ML model trained on KS EEG; small and imbalanced data deter DL	Start with feature-based SVM/RF using transfer-learning from larger epilepsy datasets Apply data-augmentation and one-class anomaly detection	Proof-of-concept classifiers; early screening tool in clinics where genetics is delayed
Longitudinal insight	Lack of serial EEG to track natural history/ treatment effect	Embed annual EEG in registries; couple with developmental scales	Allows modelling of progression, seizure remission trends; supplies endpoints for clinical trials
Cross- disorder context	Unknown if KS shares EEG signature with other chromatinopathies	Joint analyses across KS, KBG, PMS, CHD2, etc.; multivariate ML to find convergent biomarkers	Identifies common circuit deficits → broader therapeutic targets; positions KS within pan-syndromic frameworks
Multimodal integration	EEG studied in isolation; omits facial AI, genetics, iPSC networks	Fuse EEG features with facial-phenotype scores and cell-level E/I metrics via multimodal ML	Richer diagnostic models; mechanistic links from gene → cell → network → behavior

## Data Availability

This is a review, and no new data were created.

## Abbreviations

The following abbreviations are used in this manuscript:

KS	Kleefstra syndrome
ML	Machine learning
EEG	Electroencephalogram
ICA	Independent component analysis
SVM	Support vector machines
ASD	Autism spectrum disorder
CNNs	Convolutional neural networks
